# Approaches used to care for carious primary molars among pediatric dentists and general dental practitioners in Saudi Arabia

**DOI:** 10.4317/jced.54453

**Published:** 2018-03-01

**Authors:** Shatha Aldhilan, Sanaa Al-Haj Ali

**Affiliations:** 1 Department of Orthodontics and Pediatric Dentistry, college of dentistry, Qassim University, Qassim, Kingdom of Saudi Arabia

## Abstract

**Background:**

The aim of this cross sectional study was (1) to identify and compare the approaches which are used to care for carious primary molars between pediatric dentists (PDs) and general dental practitioners (GDPs) in Saudi Arabia and (2) to evaluate the knowledge level of the most appropriate treatment decisions for both groups with regard to caries in primary molars and its relation with demographic variables.

**Material and Methods:**

A random sample of 600 GDPs and all registered PDs (n = 100) in the Saudi dental society in 2016 were emailed a two part questionnaire; the first part included questions about demographic data and the second part investigated knowledge of the participants of the most appropriate treatment decision in four hypothetical case scenarios in which the severity of caries in a single primary first molar differed. Data were analyzed using chi square and ordinal logistic regression statistical tests. The significance was set at 0.05.

**Results:**

The average knowledge score was 1.28 for GDPs and 1.80 for PDs. There were significant differences between both groups on their choice of the most appropriate treatment option in three out of four scenarios. Two factors significantly improved the participants’ knowledge; age and qualification (*P*<0.050.05). PDs were more interventionists, ready to perform pulpotomy and extraction in the absence of equally appropriate treatment options.

**Conclusions:**

GDPs and PDs in Saudi Arabia had different treatment approaches of different carious conditions affecting primary molars. PDs had moderate but significantly better knowledge of the most appropriate treatment option than GDPs.

** Key words:**Dental caries, general dental practitioners, pediatric dentists, primary molars, treatment approaches.

## Introduction

Dental caries in the primary dentition is an important public health problem worldwide. In Saudi Arabia, several studies were conducted in several cities to assess the prevalence of dental caries in children with primary dentition (1-4). A recent systematic review of literature concluded that the prevalence of dental caries and its severity in children in the primary dentition in the country is high (80%) with a mean dmft of 5.0 (5). It seems that children in the Arab Gulf countries are sharing the same trend as well (6). Therefore, there is a great need for dental treatment and care among this age group of children.

Dental care for children in Saudi Arabia is provided by GDPs and PDs. PDs are well trained to provide dental care for children; however; those who are targeted to the general Saudi population are based in ministry of health facilities, universities, and the private sector. The rest of the health facilities in which specialists can be found provide care for specific Saudi subpopulations such as health facilities of the Ministry of Defense and Aviation, the Ministry of Interior, and the Saudi Arabian National Guard (7). Consequently, the availability throughout the kingdom and the accessibility to GDPs can far exceed that to specialists, which puts a big load of providing dental care for children on GDPs.

When the approaches which were used to care for primary teeth were compared between PDs and GDPs in UK, (8) and Hong Kong, (9) different opinions were observed. In one report, GDPs from UK and Japan had different treatment decisions for the same problem (10). Same finding was reported among pediatric dentists in these countries (11).

No emphasis was made in Saudi Arabia to identify and compare the approaches used in the care of primary teeth between GDPs and PDs. Therefore, the aim of this study was (1) to identify and compare the approaches which are used to care for carious primary molars between PDs and GDPs in Saudi Arabia and (2) to evaluate the knowledge level of the most appropriate treatment decisions with regard to caries in primary molars for both groups and its relation with demographic variables.

## Material and Methods

-Study sample

A simple random sample of 600 GDPs almost equally distributed across the 5 geographical regions of Saudi Arabia (central, northern, southern, eastern and western) and who represented 10 percent of registered members in the Saudi dental society in 2016 were invited to participate in this cross sectional study along with 100 (all) PDs registered in the Saudi dental society in the same year. Each selected dental practitioner was sent through email a questionnaire and a cover letter explaining the purpose of the study in the period from September-December, 2016. Practitioners willing to participate completed the questionnaire and returned it through the same email address of the principle investigator. This study was approved by the ethical committee of college of dentistry-Qassim University (reference number: EA/35/2016).

-Data collection

Participating practitioners were requested to answer a two-part questionnaire. The first part comprised biographic and demographic questions including participant’s age, gender, qualification, current practice sector, years of current practice experience, and number of child patients seen per week. The second part of the questionnaire comprised four hypothetical clinical case scenarios in which the severity of a class two carious lesion in a single first primary molar differed. The results of a qualitative study of 93 GDPs in the northwest of England also helped shape the final case scenarios. (12) Each clinical case scenario had a list of possible treatment options and participants were asked to select their single most appropriate treatment option ( Table 1). A minor modification was performed in this study to the treatment option “extraction under local anesthetic”, in which the statement” with possible construction of space maintainer” was added.

**Table 1:**
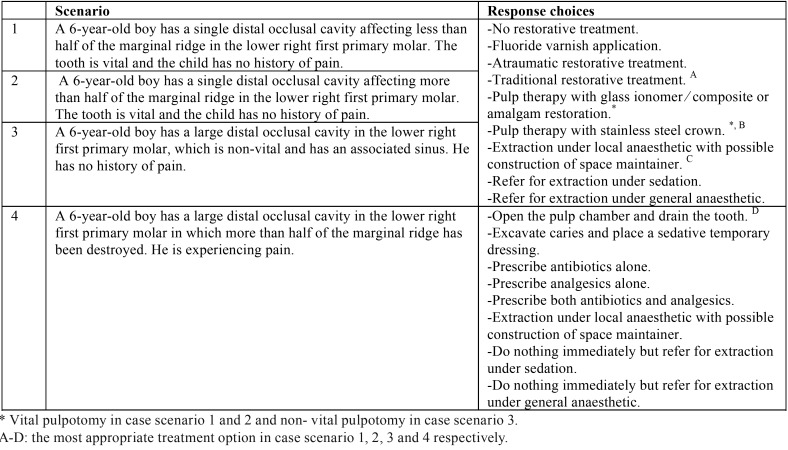
Summary of the case scenarios and the treatment options.

-Data analyses

Data was analyzed using the SPSS computer software (SPSS Version 20, Chicago, IL, USA). Simple frequency distributions of the dentists’ biographic and demographic data and the responses of each case scenario were produced. Frequency responses of the case scenarios were compared using chi square test. Participants were given a knowledge score which ranged from 0 to 4 according to their answers on part two of the questionnaire. The most appropriate answer for each case scenario equaled one point. Ordinal logistic regression was used to identify the association of dentists’ biographic and demographic profile with their overall knowledge score. The level of significance was set at 0.05.

## Results

The response rate of GDPs was 74.3% (446 out of 600) and that of PDs was 84% (84 out of 100). The average knowledge score for GDPs in part 2 of the questionnaire was 1.28 and for PDs was 1.80 (out of 4).  Table 2 shows the treatments selected by the participants in the first three case scenarios. Statistically significant differences existed between GDPs and PDs in these scenarios (*P*=0.003, 0.013 and 0.001). The majority of GDPs and PDs would restore the carious lesion in scenario one (71% vs. 66%, respectively). However, more PDs chose traditional restorative treatment when compared to GDPs (52% vs. 39% respectively). Almost half of the GDPs who chose to restore the tooth in scenario one preferred ART (31.6% vs. 38.3% chose traditional restorative treatment). More GDPs would restore the lesion in scenario two than PDs (70.9% vs. 58%) while more PDs chose pulpotomy with either a filling or a SSC when compared to GDPs (32% vs. 20.4%). Some GDPs and PDs chose fluoride varnish application in the first 2 scenarios (15.2% and 2.9% of GDPs vs. 11.9% and 3.6% of PDs in scenario 1 and 2 respectively) and few of them chose not to intervene at all. (4.3% and 3.1% of GDPs, and 6% and 3.6% of PDs in scenario 1 and 2, respectively).

**Table 2:**
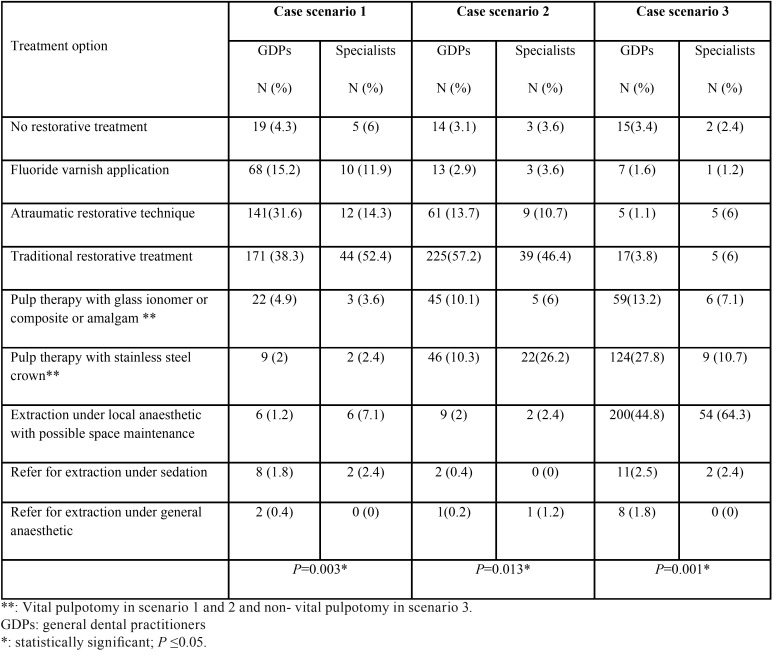
The treatments selected by the participants for case scenarios 1, 2, and 3.

In scenario three, the same tooth was non vital with an associated sinus, but the child was not in pain. The majority of PDs (64.3%) chose to extract the tooth under local anesthetic with possible construction of space maintainer compared to 44.8 % of GDPs who chose the same option. GDPs in this scenario seemed to almost equally prefer non-vital pulpotomy with a filling or SSC than PDs (41% vs. 17.8%). Very few practitioners chose a non- interventionist approach for the described tooth (3.4% of GDPs vs. 2.4% of PDs).

In scenario four, the tooth was non vital and the child was in pain. The difference in opinion between GDPs and PDs was insignificant in this case (*P* >0.05). A relatively equal proportion of GDPs and PDs chose to stabilize the tooth by opening the pulp chamber and draining the tooth (34.3% vs. 35.7%). The second option was to excavate caries and place a temporary sedative dressing (31.4% of GDPs vs. 25% of PDs) followed by the decision to extract the tooth under local anesthetic with possibly constructing a space maintainer in both groups (18.2% of GDPs vs. 20.2% of PDs) ( Table 3).

**Table 3:**
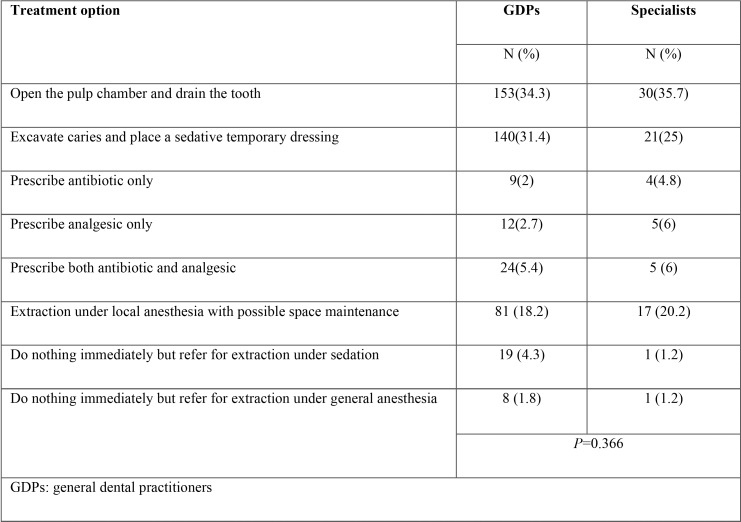
The treatments selected by the participants in case scenario 4.

 Table 4 shows the distribution of the participants according to the biographic and demographic data and the results of ordinal logistic regression analysis. Two factors had a statistically significant effect on the knowledge score of the participants; being in the middle age group (41-50 and 31-40 years) (*P*=0.009 and 0.049) and holding a specialty in pediatric dentistry (*P*=0.002); practitioners who were 41- 50 years old were 13.5 times more likely to answer the case scenarios correctly and consequently get a higher score, while those who were 31- 40 years old were 7.5 times more likely to get a higher score. On the other hand, pediatric dentists were almost twice more likely to answer the case scenarios correctly and get a higher score than general dental practitioners.

**Table 4:**
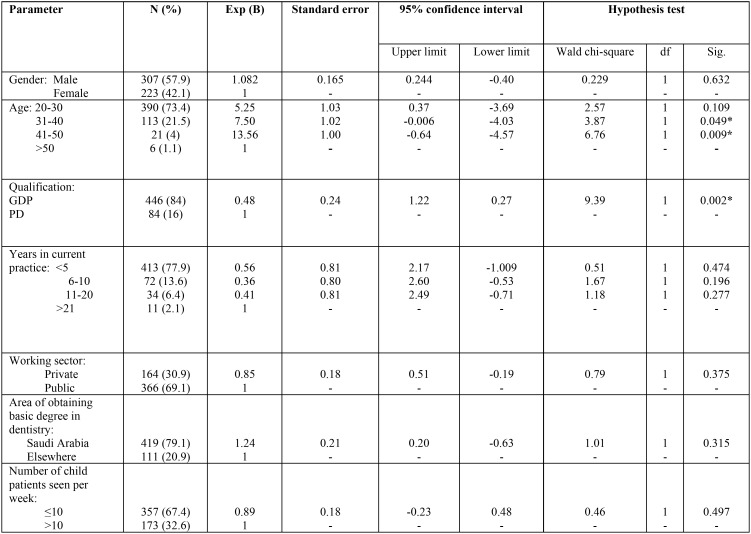
Frequency (percentage) of participants according to biographic and demographic data and association with knowledge score (* statistically significant difference).

## Discussion

This is the first study to survey the care that a national sample of dental practitioners and pediatric dentists might provide to children with carious primary molars in Saudi Arabia. Hypothetical case scenarios were used although these may not reflect actual clinical situation as some factors which may influence final diagnosis and decision making were not considered like radiographic examination of the tooth in question, financial issues and parental wishes; therefore what practitioners selected as the most appropriate treatment option is based solely on the clinical description of each case and can be different from what they actually do in practice (13). However, it is important to make sure that practitioners know the current best practice in such common conditions of the scenarios as it should always be discussed with the patient and parents before reaching a treatment decision.

In this study, the average knowledge score of Saudi PDs was better than that of GDPs (1.80 vs. 1.28 out of 4 respectively) although on a scale of zero to four, this may lie in the moderate knowledge zone which can be worrisome. The findings from the first 2 scenarios are in agreement with the findings in UK; (8) where restoration of the small lesion was preferred by the majority of practitioners; however PDs seemed to prefer traditional restorative treatment in the first scenario and traditional restorative treatment followed by pulp therapy in the second scenario, whereas GDPs tended to equally prefer ART especially in scenario one with less preference in scenario two. Therefore, in a similar clinical situation, PDs will be more likely to restore using rotary instruments or perform pulpotomy than GDPs which can be expected as specialists should be more confident in performing extensive treatment approaches regardless of the patient’s age, an explanation which was supported by previous studies in Netherlands (14,15).

In scenario three, a clear difference in treatment decision was noticed between GDPs and PDs, where PDs preferred to extract the tooth, while GDPs seemed to almost equally prefer non-vital pulpotomy with a restoration or SSC. This finding seems to contradict previous observations (8,9,11) where PDs in these countries preferred non vital pulpotomy over extraction. Few studies have suggested that pulpotomy may be performed successfully on non-vital teeth (16). However; Hill (17) found that the presence of non-vital pulp was associated with significantly reduced survival following pulpotomy in primary molars which can be related to the retained source of infection when compared with vital teeth with no evidence of extensive pulpal disease. Non vital primary teeth diagnosed with irreversible pulpitis or necrotic teeth are treated with pulpectomy or extracted with space maintenance according to the case (18). To enable comparisons with previous studies (8-11) pulpectomy was not enlisted in the treatment options in this study; therefore PDs preferred a more radical treatment approach but probably more evidence based.

In scenario four, the least disagreement in opinion was observed between both groups as opening the pulp chamber to drain the tooth and excavate caries with temporary dressing placement were listed as the most appropriate almost equally by GDPs and PDs. However, some confusion was noticed in this scenario as no treatment option was chosen by the majority of either group.

A number of dentist, patient and treatment system factors were associated with the variability in decision making according to previous studies (19). It has been stated that the personal characteristics of dentists relevant to treatment variation are skills/diligence, age/experience, knowledge, and tolerance of uncertainty (20). In this study, two factors were associated with improved knowledge; age and qualification. These factors were also associated with proper treatment decisions in Hong Kong (9). In addition, age was associated with differences in treatment choices in other fields in dentistry like prosthodontics (21,22).

The findings from this study reveal an overall variation of opinion between GDPs and PDs in Saudi Arabia and somehow moderate knowledge of the most appropriate treatment choice of carious lesions affecting primary molars. Although PDs scored better than GDPs, and they seemed more interventionists, ready to perform pulpotomy electively, and extraction under local anesthetic in the absence of equally appropriate treatment options, their knowledge score could have been better as inappropriate treatment decisions were also noticed by them. Therefore, more emphasis on the current best evidence available from pediatric dentistry textbooks and conference proceedings should be placed. Recent clinical guidelines of the American academy of pediatric dentistry are better enrolled along with textbooks in the curricula of undergraduate and postgraduate students specializing in Pediatric dentistry in Saudi Arabia. The Saudi dental society is requested to place more emphasis on its members through lectures especially demonstrators and alumni as these are expected to be more willing to implement new effective treatment approaches and discard established treatments with poor effectiveness.
